# Mobile Data Gathering and Preliminary Analysis for the Functional Reach Test

**DOI:** 10.3390/s24041301

**Published:** 2024-02-17

**Authors:** Luís Francisco, João Duarte, Carlos Albuquerque, Daniel Albuquerque, Ivan Miguel Pires, Paulo Jorge Coelho

**Affiliations:** 1 Electrotechnical Department, Polytechnic University of Leiria, 2411-901 Leiria, Portugal; 2 Health Sciences Research Unit: Nursing (UICISA: E), Nursing School of Coimbra (ESEnfC), 3004-011 Coimbra, Portugal; calbuquerque@essv.ipv.pt; 3 Higher School of Health, Polytechnic Institute of Viseu, 3504-510 Viseu, Portugal; 4 Child Studies Research Center (CIEC), University of Minho, 4710-057 Braga, Portugal; 5 Instituto de Telecomunicações, Escola Superior de Tecnologia e Gestão de Águeda, Universidade de Aveiro, 3750-127 Águeda, Portugal; dfa@ua.pt (D.A.); impires@ua.pt (I.M.P.); 6 Institute for Systems Engineering and Computers at Coimbra (INESC Coimbra), 3030-290 Coimbra, Portugal

**Keywords:** functional reach test, smart wearables, inertial sensors, monitoring apps

## Abstract

The functional reach test (FRT) is a clinical tool used to evaluate dynamic balance and fall risk in older adults and those with certain neurological diseases. It provides crucial information for developing rehabilitation programs to improve balance and reduce fall risk. This paper aims to describe a new tool to gather and analyze the data from inertial sensors to allow automation and increased reliability in the future by removing practitioner bias and facilitating the FRT procedure. A new tool for gathering and analyzing data from inertial sensors has been developed to remove practitioner bias and streamline the FRT procedure. The study involved 54 senior citizens using smartphones with sensors to execute FRT. The methods included using a mobile app to gather data, using sensor-fusion algorithms like the Madgwick algorithm to estimate orientation, and attempting to estimate location by twice integrating accelerometer data. However, accurate position estimation was difficult, highlighting the need for more research and development. The study highlights the benefits and drawbacks of automated balance assessment testing with mobile device sensors, highlighting the potential of technology to enhance conventional health evaluations.

## 1. Introduction

Smart wearables are ubiquitous in daily life. The number of connected wearable devices worldwide rose from 325 in 2016 to 722 million in 2019, with the last perspectives pointing to more than 1000 million in 2022 [1]. These devices seamlessly integrate technology into routine items, such as clothing, watches, and glasses, providing an exceptional means of extracting data from individuals’ everyday lives.

Beyond daily use, smart wearables present tremendous potential for medical applications, especially regarding follow-up approaches and simple metric extraction, with minimal impact on individuals’ lives [2]. The most popular devices in health research include fitness trackers and wearables with accelerometers. Typical measurements include steps, heart rate, sleep duration, and—less frequently—blood pressure, skin temperature, oximetry, and respiratory rate [3]. Among the potential applications of smart-wearables in medicine/health, balance-related systems have been those with faster impact, such as balance analysis, gait analysis, and fall detection [4,5,6]. These systems take advantage of the sensors present in smart wearables to collect data and process it to extract valuable medical information, usually motion-related information.

Older adults are the ones that most benefit from the development of such systems since this age group is often associated with balance loss. It is estimated that 26.5% of individuals above 65 years old fall annually [7]. These incidents had an impact of USD 50 billion (between fatal and nonfatal falls) in 2015 in the United States of America alone [8]. Recent studies suggested that fall prevention programs, conjugating functional training with gait and balance assessment screening, can significantly reduce the rate and the number of falls and, consequently, the related burden for healthcare systems [9,10]. Standard tests used to assess functional balance include Berg’s balance scale (BBS), timed up and go (TUG), and functional reach test (FRT) [10,11,12].

**Figure 1:**
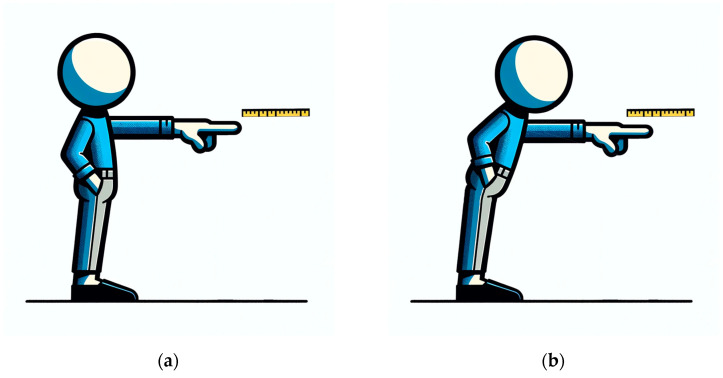
Example of the conventional FRT balance test. (**a**) Starting position for the procedure; (**b**) final position without losing balance.

The FRT is a quick single-task dynamic test allowing reliable balance evaluation. Typically, a scale (often a graduated ruler) is mounted horizontally at shoulder height on a wall to measure the FRT distance. The dominant arm should be extended at a 90° angle toward the body by the individual. The starting point of the ruler, or the scale’s reference point, is where the fingers should be placed. The person should incline/lean/bend their torso forward, just turning their hips to the maximum extent without elevating their ankles, as depicted in Figure 1. Additionally, recent reports suggest that wearable sensors may increase diagnostic accuracy while allowing movement recording and recognition [13]. Recently, a modification of the FRT has been proposed for sitting balance evaluation in stroke-affected patients [13,14].

Despite the worldwide efforts to evaluate the causes and prevent balance-related burdens, the methodologies used in balance evaluation are generally time-consuming; present lower-than-desired accuracy, sensitivity, and specificity; and ultimately lack predictive ability [15,16].

The paper’s relevance aligns with the ambient assisted living (AAL) trend, which advocates using artificial intelligence (AI) in new services, products, and processes that help to provide safe, high-quality, and independent lives for fragile and older adults. These innovative tools engage older adults in managing their health and fitness data collected on the individual’s smartphone or wearable devices, providing a more comprehensive overview of an individual’s physical condition and progress. This paper focuses on the research and development of a new tool for balance assessment, taking advantage of the data extracted from inertial sensors from the widely available smart wearables, especially smartphones. The mobile device orientation should be estimated through a sensor fusion algorithm, followed by an estimate of the respective position. The FRT metric should be hereafter computed based on the position-estimated data.

## 2. Related Work

Smart wearables integrate electronic technology—namely microprocessors, batteries, and sensors—into clothing and accessories such as watches, bracelets, and rings. Smart wearables allow data collection from sensor arrays placed near the body. Currently, these devices carry onboard sensors—such as inertial measurement units (IMU) and magnetic, angular rate, and gravity (MARG) sensor arrays—that can gather motion-related data. Smart wearable data have been used in several medical applications, including physiological signals monitoring and anatomical movement monitoring [17]. These data can be used for several objectives to estimate the FRT metric, one of the most used methodologies for balance assessment in older adults. This work intends to use IMU and MARG data from mobile devices to estimate the FRT metric.

### 2.1. Smart Wearables Data in Medical Applications

The level of balance of older adults and individuals with cardiovascular, respiratory, and motor diseases has increased in importance for study by the scientific community. This concern is because these individuals risk falling due to their injuries and/or limitations. It is estimated that 1/3 (one-third) of individuals aged over 65 years old fall each year. Fall-related injuries represent an increased burden for healthcare systems worldwide. These injuries usually come from fractures and head injuries, reduced life quality, fear of falling, loss of confidence, and self-restricted activity levels, leading to reduced physical function and social interactions. This starts a snowball effect since the lack of physical activity is related to increased fall risk.

Different studies demonstrated that physical exercise is related to decreased risk of falls in older adults by approximately 25%. Moreover, implementing physical exercise programs among the elderly population has shown to be cost-effective considering the burden of fall-related injuries. The benefits of physical exercise were demonstrated when applied in groups or individually, mainly if targeted to balance training. The FRT is valuable in assessing an individual’s balance and stability.

Numerous existing tests measure individuals’ body balance, such as the postural stress test (PST), the center of pressure excursion (COPE), the platform perturbation test (PPT), the Berg balance scale (BBS), timed up and go (TUG), and the computerized dynamic posturography (CDP). The PST uses an ordinal point measurement, which implies that it is not a continuous measurement test, so the test’s sensitivity is lower. The COPE and the PPT require sophisticated laboratory equipment, and both tests assess responses to external stimuli [13,18,19,20]. The TUG is a timed test that measures, in seconds, the time taken by an individual to get up from a chair with a backrest and armrests (where the seat has an approximate height of 46 cm), walk about three meters, turn around, walk back to the chair, and sit down again. The TUG must be performed without physical aids (stretches, walkers) or human assistance [21]. The BBS is a scale of 14 different tasks/tests evaluated by directly observing performance. Task execution time requires 10 to 20 min. Tasks are evaluated from 0 to 4, with 0 being non-resolvable and 4 being solvable without the need for help. Static and dynamic aspects can be evaluated depending on the score achieved, as the score ranges are: (i) 0 to 20 represent a low balance, (ii) 21 to 40 represent an acceptable balance, and (iii) 41 to 56 represent a good balance [22].

### 2.2. Functional Reach Test

FRT, first proposed by Duncan et al. in 1990, measures the distance a person can reach while standing. The FRT offers insights into a person’s ability to perform activities safely and independently. The values in Table 1 are the FRT reference (norm) values, considering age and sex (male and female), obtained by Duncan et al. [18].

Based on the test results, healthcare professionals can implement tailored procedures to improve the patient’s balance and stability, hoping for a better quality of life [18].

The FRT was designed to be a functional, effective, and easy-to-perform test with the advantage that the cost of carrying out the test is affordable compared to other tests. The primary purpose of the FRT is to assess an individual’s functional reach and ability to maintain balance during movement. The FRT allows healthcare professionals to determine the level of independence and the need for interventions to improve the patient’s stability and balance. This test provides valuable information about a person’s ability to reach objects or perform specific movements while maintaining balance.

**Figure 2:**
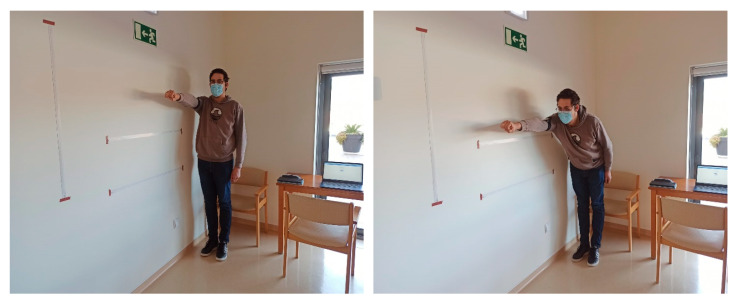
Illustrative image of the execution of the FRT by one of the authors.

During FRT, as represented in Figure 2, the participant stands upright with feet slightly apart. They are instructed to extend their dominant arm forward and reach as far as possible without losing their balance or moving their feet. The achieved distance is measured and recorded [18].

Based on the distance covered during the FRT, the person may be more or less at risk of falling (a person at a high risk of falling is named a positive test). Considering the distance reached, recent motion-based studies adopted the measurements for the risk of falling. It is classified with the following data [13]:

- If the reach distance exceeds 25.40 cm, it is considered a negative test and a low risk of falling.
- If the reach distance is within the range of 15.24–25.40 cm, the risk of falling is twice as high during the next six months.
- If the reach distance is less than 15.24 cm, there is a four times greater risk of falling during the next six months.

The authors in [13] observed that simple range tasks represent the same control center pressure excursion within the base of support as learning tasks examined traditionally with COPE. Just as COPE deteriorates with advancing age, an individual’s functional range can also deteriorate, thus serving as a protective mechanism to minimize disturbance of the center of gravity and prevent falls.

The FRT assesses nervous system function, postural stability, and the ability to reach functionality in daily activities. It is a valuable instrument for monitoring elderly populations or individuals with neuromuscular disorders that may affect balance, as it provides objective information about an individual’s balance and stability during a functional reaching movement [18].

Some relevant works in this scientific area are presented, and will be grouped by the various conditioning factors (diseases and age). Studies on people who have suffered a stroke are essential given the great relevance and number of studies, with studies on the elderly population presented in Section 2.2.2 and on other diseases in Section 2.2.3.

#### 2.2.1. FRT Related to Stroke

The realm of stroke rehabilitation research is marked by innovative approaches and diverse methodologies, as evidenced in [13,14,23,24,25,26,27]. De Luca et al. [23] utilized a robot-assisted exoskeleton in a study involving 16 chronic stroke survivors, focusing on improved postural control and motor skills through enhanced spine alignment and intersegmental coordination. Caimmi et al. [24] explored a similar technology with 19 participants, assessing the viability and efficacy of a robotic exoskeleton in aiding upper limb movements against gravity, emphasizing safety, tolerability, and impacts on quality of life and motor performance. In contrast, Williams et al. [28] developed the mStroke system, a mobile-app-based solution with wireless body motion sensors to assess fall risk, validated through a study on healthy adults. Fishbein et al.’s study [25] with 22 participants compared virtual-reality-based dual-task training to conventional single-task training, demonstrating potential improvements in balance and walking for post-stroke patients. Bruyneel et al. [26] investigated center of pressure measurements in 32 stroke survivors during an unstable sitting test to assess trunk control, integrating various assessments to validate COP measures. Marchesi et al. [14] focused on upper body kinematics and muscular activity in 15 chronic stroke survivors performing a modifiedFRT, aiming to understand biomechanical and neuromuscular mechanisms behind balance. Fell et al. [27] promoted the mStroke mobile health system, with their study involving 35 post-stroke individuals emphasizing the importance of long-term support and monitoring for stroke recovery. Each study contributes uniquely to the field, ranging from robotic assistance, virtual reality training, and mobile health applications to biomechanical assessments, collectively enriching our understanding and capabilities in stroke rehabilitation.

#### 2.2.2. FRT Related to Older Adults

Hsiao et al. [29] and Mengarelli et al. [30] both focused on the use of video game technology for balance assessment in older adults, with Hsiao et al. exploring the Microsoft Kinect system and Mengarelli et al. investigating the Nintendo Wii Balance Board (WBB). While Hsiao et al. emphasized the Kinect’s potential as a cost-effective and non-invasive tool for individualized fitness interventions, Mengarelli et al. validated the reliability of the WBB against a gold-standard force platform. Moriyama et al. [31] diverged by analyzing movement patterns during the FRT in young and older adults, concluding that these patterns are unreliable predictors of physical function. Ghahramani et al. [32] also utilized the FRT but focus on the coordination between the chest and pelvis as indicators of balance deficits in older persons. In contrast, Chen et al. [33] aimed to integrate various physical performance tests into a computerized frailty assessment tool, the FAT system, for evaluating and monitoring frailty progression in older people. Bao et al. [34] examined the effectiveness of balance training with and without sensory augmentation in healthy, community-dwelling older adults, demonstrating the feasibility of using sensory augmentation devices for balance rehabilitation.

#### 2.2.3. FRT Related to Other Conditions

Comparing the studies [35,36,37,38,39,40,41,42,43], we observe a diverse range of approaches and focuses in assessing postural control and balance. Dewar et al. [35] and Santamaria et al. [36] both concentrated on children with cerebral palsy (CP) yet with different objectives: Dewar et al. [35] evaluated clinical criteria for FRT using kinematic markers, whereas Santamaria et al. [36] explored the effectiveness of the trunk support trainer (TST), a robotic device, in promoting independent sitting. On the other hand, Reguera-García et al. [37] and Tanaka et al. [38] used advanced technologies like pressure-mapping and motion-capture systems but for different populations and purposes: the former assessed postural control in seated patients with various neurological conditions, while the latter compared motion capture systems in identifying movement strategies during the FRT in young, healthy individuals. Nozu et al. [39] and Verdini et al. [40] both studied healthy participants with distinct focuses: Nozu et al. [39] examined the impact of somatosensory disturbances on balance, and Verdini et al. [40] validated the Nintendo Wii Balance Board as a tool for measuring force during balance tests. In a similar vein, Son et al. [41] and Park et al. [42] investigated specific conditions affecting balance and mobility; Son et al. [41] assessed the impact of personal protective equipment on firefighters’ mobility, while Park et al. [42] explored how starting position influences reach distance and center of pressure movement in healthy males. Finally, Ayed et al. [43] broke new ground by demonstrating the feasibility of remotely assessing balance using the Microsoft Kinect v2 sensor, highlighting potential benefits for patients with limited access to medical facilities.

### 2.3. Orientation Estimation

Microelectromechanical systems (MEMS) consist of microdimensional devices that combine mechanical and electrical elements [44]. Integrating silicon MEMS and complementary metal oxide semiconductor (CMOS) technology and production cost reduction promoted the rapid integration of MEMS/CMOS systems in a wide range of consumer electronic products [45].

Orientation estimation using MEMS entails measuring and computing an object’s orientation using small sensors and devices [46]. It is essential in many industries, including consumer electronics, robotics, aerospace, and automotive systems [46]. MEMS devices integrate electronics and mechanical parts, like sensors and actuators, onto a single silicon chip [47]. Accelerometers and gyroscopes are the most often utilized MEMS devices in orientation estimation.

MEMS accelerometers measure linear acceleration to determine device orientation, detecting gravity’s impact on an object’s orientation relative to the Earth’s surface, such as in smartphones [48]. MEMS gyroscopes measure angular velocity, crucial for understanding an object’s speed and direction, correcting and stabilizing orientation [49].

The data from these MEMS sensors is frequently integrated and processed using techniques like Kalman filters or complementary filters for efficient orientation estimation [46].

#### 2.3.1. Coordinate Frames

The sensor orientation can be described with respect to a reference frame. Thus, the attitude of the sensor is described by a rotation of the sensor frame, or body frame, concerning the reference frame [50]. Different coordinate systems can be used, including geodetic coordinates, Earth-Centred Earth-Fixed (ECEF) coordinates, Azimuth-Elevation-Range (AER) coordinates, and East-North-Up (ENU) coordinates [51].

In applications that consider a small area (usually in distances within 4 km), and the curvature of the Earth can be assumed as a flat surface, the local ENU coordinate system is often used since it uses the intuitive Cartesian coordinate system, allowing the use of Euclidean geometry. In this system, the x-axis points east, the y-axis points to geomagnetic north, and the z-axis has the same direction of the gravity vector, pointing upwards (outwards) regarding the Earth’s center. In the present paper, the ENU coordinate system is considered the reference frame, also named the Earth frame.

#### 2.3.2. Orientation Representation

An object’s orientation in ℝ^2^ can be represented as a single angle since rotations in ℝ^2^ have their axis of rotation perpendicular to the *XOY* plane [52]. Representing an object’s orientation in three-dimensional space is more complex since in ℝ^3^, the axis of rotation can have any spatial orientation (main axes in three-dimensional space—*Ox*, *Oy*, *Oz*). Rotations in ℝ^3^ can be defined from rotation matrices, Euler angles, axis-angles, and quaternions. Euler angles are used in biomechanics to describe the orientation of a body segment or object in three-dimensional space, typically involving rotations around three principal axes (X, Y, and Z)—often referred to as Roll, pitch, and yaw. These angles are crucial for quantifying the motion of joints and segments during movement. We refer the reader to some references to obtain more concrete details about the various representations of the angles and their respective conversions [53,54,55]. Nevertheless, the main advantages and disadvantages of each coordinate system are described in Table 2.

Quaternions provide a straightforward method for estimating a system orientation based on gyroscope data. The angular rate of the sensor, *ω*(*θ*), is given by Equation (1):

(1) $$\omega (\theta )=\frac{d\theta }{dt}$$

The gyroscope’s angular rate can be integrated into a time interval (*t*) and a sampling period (*T_s_*). The sum of each measurement will return an absolute angle *φ*. The angular velocity integration is given by Equation (2):

(2) $$\varphi =\int_{0}^{t}\omega (\theta )dt=\sum_{0}^{t}\omega (\theta )dt\cdot T_{s}$$

With a set of data collected by the gyroscope, it is possible to create a quaternion-based matrix (Ω), given by Equation (3):

(3) $$\Omega =\left[\begin{array}{cccc} 0 & -\omega_{x} & -\omega_{y} & -\omega_{z}\\ \omega_{x} & 0 & \omega_{z} & {-\omega }_{y}\\ \omega_{y} & -\omega_{z} & 0 & \omega_{x}\\ \omega_{z} & \omega_{y} & -\omega_{x} & 0 \end{array}\right]$$

From the data of a quaternion q at time *t* − 1 and with a given angular velocity, it is possible to estimate the quaternion at time t. The quaternion qt revolved by the gyroscope, the antecedent attitude quaternion *q_t_*_−1_ is multiplied by Ω, then half of the period *T_s_*, as presented in Equation (4) [56]:

(4) $$q_{t}=q_{t-1}\cdot Ω\cdot \frac{1}{2}\cdot T_{s}$$

#### 2.3.3. Attitude and Heading Reference System

An Attitude and Heading Reference System (AHRS) estimates an object’s orientation [57]. These systems are based on IMU or MARG sensor array data and—through the implementation of a fusion algorithm, usually a Kalman filter—determine the roll, ϕ; pitch, θ; and yaw, ψ (Tait–Bryan angles) [58]. Kalman-filter-based AHRS algorithms are accurate and effective because they allow compensation for gyroscope drift using gravity and Earth’s magnetic field vectors through sensor fusion [59,60,61]. However, the implementation can be complicated, requiring high sampling rates, large state vectors, and an Extended Kalman filter (EKF) implementation to linearize the problem [57,59,60,61]. Moreover, Kalman filter-based algorithms usually involve large data requirements and computational load. Some approaches have been proposed as an alternative to using the Kalman filter—in particular, complementary filtering [62], which is effective at a low computational cost, and optimization algorithms using quaternion algebra, which provide accuracy equivalent to Kalman-filter-based approaches at low computational cost and reduced frequencies, which is ideal for small wearable devices [57].

#### 2.3.4. Sensor Fusion

The availability of a wide range of sensors, in number and typology, led to the fast development of sensor fusion research. These algorithms present the ability to combine information from different data sources, leading to (i) higher resolution of the data, (ii) increased certainty or confidence rate associated with improved signal-to-noise ratio and information redundancy, (iii) increased accuracy, and (iv) completeness since different sensors provide different insights resulting a broader view. However, sensor fusion presents some challenges, including (i) inappropriate sensor registration, i.e., the integration of multiple sensor data considering a common referential and the treatment of individual errors related to each one, (ii) uncertainty due to the presence of confliction data resulting in noise and ambiguity, (iii) incomplete, inconsistent, and spurious data, related to incorrect sensor registration or sensors inconsistency, (iv) track-to-track association, i.e., data match between different sensors (e.g., each sensor represent the same object), (iv) granularity, due to different sparsity of different sensors data, and (v) unsynchronized time scales due to different sampling rates of the sensors [63].

Ideally, orientation estimation would be possible using the data of a single gyroscope. However, gyroscope data is associated with drift. Thus, IMU sensor array data fusion surged to correct gyroscope-based estimates. Accelerometer and magnetometer data can evaluate attitude while correcting the presence of magnetic disturbance. The most used sensor fusion algorithms include the Kalman filter, complementary filter, optimization algorithms, and—more recently—neural-networks-based algorithms.

Based on the use of Kalman-filter-based sensor fusion algorithms, it includes two different fusion approaches: state-vector fusion and measurement fusion. Kalman filters are based on recursive Bayesian filtering, assuming Gaussian noise, and are appropriate for linear systems [64]. An extended Kalman filter (EKF) is commonly used in nonlinear systems.

Complementary filtering is a more straightforward approach, thus resulting in a less computationally demanding approach [65]. Complementary filtering considers two sensor measurements, one composed of high-frequency signals and the other composed of low-frequency signals. Complementary filtering in AHRS using IMU and MARG sensor arrays considers the accelerometer and gyroscope data as the primary sensors. When available, the magnetometer data is used as a correction sensor. Accelerometer data is subjected to a low pass filter, such as a moving average filter, since it considers the forces (inertial) acting on the sensor, and the high-frequency components are related to disturbances, namely vibrations and centripetal forces. On the other hand, the gyroscope is subjected to a high pass filter to remove drift related to sensor integration while maintaining short-term data, which is precise. One of the most implemented algorithms in practice is a complementary filter first proposed by Mahony, suggesting an adaptive gain optimization method to determine the gain parameters inherent to the complementary filtering.

Madgwick proposed another successful sensor fusion algorithm for AHRS [66]. This algorithm uses a gradient descent optimization algorithm computed from accelerometer data considering a predefined gravity vector and, optionally, from magnetometer data considering a predefined Earth magnetic field vector to correct gyroscope integration drift/accumulative error. A modification to this algorithm was later proposed by Kok, fastening the convergent of the gradient descent algorithm and reducing the AHRS algorithm complexity [67]. Recently, Madgwick proposed an improvement to the original algorithm and an extended complementary filter based on the algorithm proposed by Mahony [68]. The extended complementary filter algorithm considers the gravity component and the geomagnetic north vector for attitude correction [69]. The gyroscope data correction disables this information depending on whether dynamic motion or magnetic disturbances are detected [70].

### 2.4. Position-Estimation Algorithms

Position and orientation estimation is important in many fields, including aerospace, robotics, navigation, machine interaction, and human motion analysis [57,71]. In clinical areas, such as rehabilitation and elderly monitoring, motion tracking systems can be a valuable technology for continuous monitoring [72]. However, most methods have significant shortcomings regarding real-time operation, wireless properties, data correctness, and portability [73]. In particular, FRT is a manual method that produces subjective results depending on the operator [74].

We propose a new automated FRT measurement tool based on MEMS in widely used smartphones and wearables [71]. MEMS allow tracking of rotational and translational motions due to triaxial accelerometers, gyroscopes, and/or magnetometers. The primary concerns for these approaches are due to the accelerometers and gyroscopes, which can accumulate significant drift over time, leading to inaccuracies in position estimation due to the accumulation of noise due to double integration. Magnetometers are sensitive to nearby magnetic fields, which can distort heading information. As mentioned previously, these limitations often necessitate complex filtering algorithms, such as extended Kalman filters, to mitigate errors and improve accuracy. Even with advanced processing, the precision of position estimates from these sensors alone cannot match that of GPS or other external positioning systems, especially over longer durations or in environments with high magnetic interference or limited movement. Nevertheless, in this particular application, for FRT measurements, GPS systems are inadequate due to the low movement span and because most of these tests are performed indoors.

## 3. Materials and Methods

### 3.1. FRT Data Collection through a Mobile App

Sensory data was gathered from the smartphone’s sensors (Xiaomi Redmi Note 8 Pro M1906G7G, equipped with Android version 11 RP1A.200720.011). The device was placed on the arm of the subjects with a smartphone armband. The manual method, proposed by Duncan et al. [18], was used as a control to obtain gold-standard measurements for methodology comparison purposes.

The data were collected using a mobile app developed in Java for Android-based devices (Android 5.0 SDK 21 and above) [75]. The app, named Wearables Balance, concerning the project’s objective—to evaluate the balance in older adults—and the means—wearable devices—were developed in the Android Studio IDE (Android Studio Dolphin|2021.3.1 Patch 1). The app presents both collection center and subject structures, allowing users to register data related to both, such as the region of the collection center, the subject’s weight and height, and the assessment date. The data can also be shared via the smartphone in a text file to, for example, a cloud service. Wearables Balance uses tools provided by a Google-supported app development platform named Firebase. Firebase provides:

- Firebase Authentication—an authentication platform that implements a simple and secure authentication system;
- Cloud Firestore—a NoSQL document database where the App’s data structures (users, collection centers, and subjects) are stored, allowing simple data querying;
- Cloud Storage for Firebase is a cloud storage service that allows uploading the collected data (wearable sensor data) in a text file format.

The Wearables Balance app collects data from the device using the classes in the “android.hardware” package that provides access to the device’s sensor data (Sensor, SensorManager, and SensorEventListener). The tool allows the collection of the following sensor and sensor-derived data: (i) accelerometer, both calibrated and uncalibrated (m∙s^−2^); (ii) gyroscope, both calibrated and uncalibrated (rad∙s^−1^); (iii) magnetometer, both calibrated and uncalibrated (μT); (iv) gravity (m∙s^−2^); (v) linear acceleration (m∙s^−2^); and (vi) rotation vector (a quaternion representation of the orientation). The timestamp of each recorded sample will be obtained using (i) the global system function, which provides millisecond precision; and (ii) the variable timestamp from the SensorEvent class, which provides nanosecond precision. The acquisition rate is defined as the fastest possible, configuring the class SensorManager property Sensor_Delay_Fastest. These configurations resulted in an acquisition rate of 400 Hz for the accelerometer and gyroscope data and 100 Hz for the magnetometer data.

To perform a valid test, some relevant individual data—namely weight, height, and age—are collected and introduced in the Wearables Balance app. Afterward, the sensors’ value-recording starts, and the smartphone used to collect sensory data is placed laterally on the upper arm of the dominant side of the individual using an armband. Then, the participant performs the three attempts for FRT, after which the recording will be stopped. The recorded data are immediately uploaded to the cloud after the test.

### 3.2. Functional Reach Test Distance Estimation

#### 3.2.1. Orientation Estimation

Different AHRS sensor-fusion algorithms were considered to obtain the orientation estimation: (i) complementary filter; (ii) EKF; (iii) Mahony algorithm; and (iv) Madgwick algorithm. These algorithms were analyzed after data acquisition using the Python AHRS toolbox. The AHRS toolbox makes available the implementation of the most used/known algorithms and methods for attitude estimation. The available algorithms were benchmarked against Android’s software-based estimate of the linear acceleration (excluding gravity).

Three statistical evaluation metrics were considered: (i) the mean absolute error (*MAE*); (ii) the mean squared error (*MSE*); and (iii) the root mean squared error (*RMSE*).

The *MAE* quantifies the average of the absolute differences between the estimated and actual values in two time series. The *MAE* is computed as Equation (5), where *n* is the number of samples, *y_i_* is the prediction, and *x_i_* is the truth value.

(5) $$MAE=\frac{\sum_{i=1}^{n}\left|y_{i}-x_{i}\right|}{n}$$

The *MSE* measures the average squared error between paired observations in a two-time series. The *RMSE* consists of the root of the *MSE* and is widely used as a loss function in a model or estimator. The *MSE* and *RMSE* are computed as Equations (6) and (7).

(6) $$MSE=\frac{\sum_{i=1}^{n}{\left(y_{i}-x_{i}\right)}^{2}}{n}$$

(7) $$RMSE=\sqrt{\frac{\sum_{i=1}^{n}{\left(y_{i}-x_{i}\right)}^{2}}{n}}$$

#### 3.2.2. Real-Time Orientation Estimation

**Figure 3:**
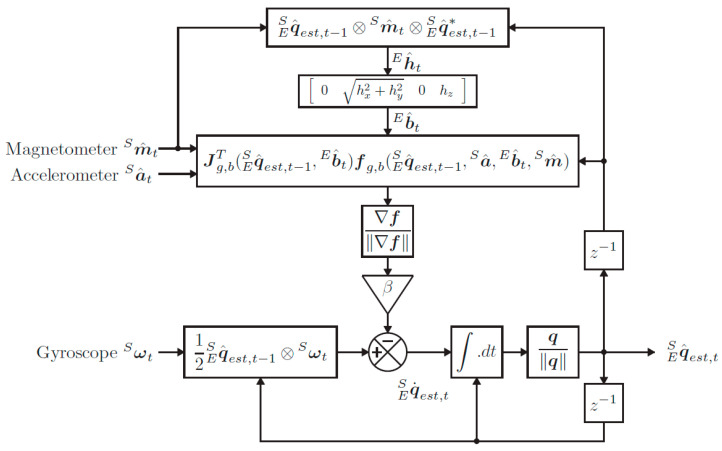
Sensor fusion using MARG sensor arrays [57].

The proposed orientation estimation algorithm is based on the method described by Madgwick et al. [57]. Madgwick’s algorithm implements a gradient descent fusion algorithm that determines a quaternion estimation of the orientation from the gyroscope data that is corrected using a quaternion resulting from accelerometer and magnetometer data. This algorithm provides attitude estimation accuracy equivalent to Kalman-filter-based approaches at low computation cost while limiting the effect of local magnetic disturbances. The fusion algorithm process is schematized in Figure 3.

The triaxial sensors measurement can be represented as a quaternion with a null scalar component, as given in Equation (8). The superscript S denotes that the measurements are with respect to the sensor frame. Linear acceleration, angular velocity, and geomagnetic field strength measurements are represented as aS, ωS and mS, respectively.

(8) s S=0 sx sy sz

At time *t*, the orientation of the Earth frame relative to the sensor frame, qω, tES, is given by the numerical integration of the quaternion derivative, where the initial conditions are known, as described in Equations (9) and (10):

(9) q˙ω,tES=12q^est,t−1ES⊗ωt S

(10) qω,tES=q^est,t−1ES+q˙ω,tESΔt

q^est,t−1ES is the prior orientation estimate, with the ^ accent denoting a normalized quaternion. ωtS refers to the gyroscope output at time *t*. Δ*t* is the sampling period. The quaternion derivative q˙ω,tES relates the time derivative of the quaternion with angular velocity, as denoted by the subscript *ω*, indicating that the quaternion derivative is calculated from angular rates.

Madgwick et al. proposed an optimization problem to find the quaternion that represents the rotation that aligns a predefined reference direction in the Earth frame, d^E, with the corresponding measured direction in the sensor frame, s^S [57]. The optimization problem is defined as Equation (11), and the corresponding objective function is defined as Equation (12):

(11) minq^ES∈R4⁡fq^ES,d^E,s^S

(12) fq^ES,d^E,s^S=q^*ES⊗d^E⊗q^ES−s^S

The algorithm uses the triaxial accelerometer data to obtain a predefined reference direction in the Earth frame, a^tS, as a reference for the gravity vector, g^E, which defines the vertical z-axis, given by Equation (13):

(13) g^E=0 0 0 1

When considering MARG sensor arrays, the triaxial magnetometer data, m^S, is also used as a reference for the Earth’s magnetic field vector, b^E, that presents components in one horizontal axis and the vertical axis, represented by Equation (14):

(14) b^E=0 bx 0 bz

To account for soft iron interference impacting the Earth’s frame, the Earth’s magnetic field direction in the Earth frame, at time *t*, h^tE, is computed as presented in Equation (15):

(15) h^tE=0 hx hy hz=q^est,t−1ES⊗m^tS⊗q^est,t−1*ES

The reference direction of the Earth’s magnetic field, at time *t*, b^tE, can be derived from the normalization of h^tE in the x and z axes of the Earth frame, as shown in Equation (16):

(16) b^tE=0 hx2+hy2 0 hz

A gradient descent algorithm was proposed to solve the minimization problem and compute the quaternion, as denoted by the subscript ∇. Accordingly, the estimated orientation is given by Equation (17):

(17) q∇,tES=q^est,t−1ES−μt∇f∇f

The use of a fusion algorithm accounts for the frequency of errors in q∇,tES through the orientation estimate qω,tES, and compensate for the corresponding integral drift in qω,tES through q∇,tES while ensuring convergence from the initial conditions. Madgwick et al. proposed the computation of one iteration per time sample to improve computational performance. In this case, the rate of convergence of the orientation estimate should be larger than the rate of change of the physical orientation.

#### 3.2.3. Distance Estimation

After obtaining an orientation estimate, the accelerometer data is rotated through the quaternions and the gravity component (9.81 m∙s^−2^) to yield acceleration in the Earth frame, a^E, as described in Equation (18):

(18) a^ E=qa Sq*−9.81g^ E

Afterward, the acceleration is integrated to yield linear velocity (m∙s^−1^) and integrated again to yield position (m). After each integration, the data pass through a high-pass Butterworth filter to remove drift (cutoff = 0.01 Hz, order = 5). Then, the main direction of the movement in the XOY plane (parallel to the floor) and the distance corresponding to the FRT is determined, consisting of the distance between the extreme points of the movement.

## 4. Results and Discussion

### 4.1. Data Collection Mobile Application

The automatization of balance assessment through the FRT is intended to be simple, fast, and reliable. Thus, the design of the data collection mobile application—Wearables Balance—must fulfill some characteristics based on the literature.

#### 4.1.1. Properties of the App

The Wearables Balance application was developed for Android devices using Android Studio IDE, version Dolphin (2021.3.1) with the following requirements:

- Programming language: Java.
- Minimum SDK: Android 5.0 (Lollipop).
- Communication with other mobile devices: Bluetooth.
- Sensors to collect: accelerometer, gyroscope, and magnetometer.
- Data storage via Google LLC’s Firebase services.
- Support two languages: English and Portuguese.

#### 4.1.2. Data Structures

**Figure 4:**
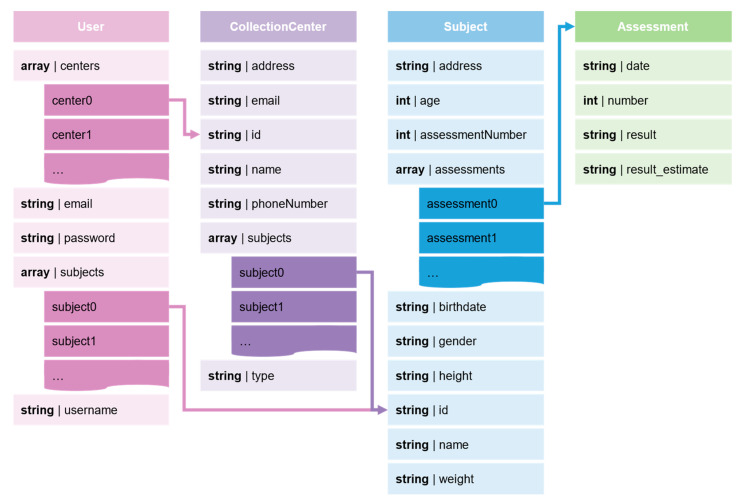
Data structures of the Wearables Balance app.

Four types of data structures were developed to facilitate the organization of the collected data: User, CollectionCenter, and Subject (and Assessment), as shown in Figure 4.

The User class is intended to store the collection centers and subjects the user can access. The CollectionCenter class allows users to gather the collection center’s name, address, contact information (email, phone number), and type of collection center. This class also contemplates an array of strings that stores the related subjects (via the identifier—id), allowing users to group subjects into collection centers. The Subject class stores subject demographic data (age, gender, weight, and height) and considers an array to store the assessment data. The assessment data consists of a structure storing the assessment date, the number of assessments (a count of the assessments registered in the subject), the assessment result obtained via the manual method, and the resulting estimate obtained through the proposed method. Note that the results are stored in a string format to allow the registration of multiple trials in the same assessment. These data are stored in the Firestore Database.

#### 4.1.3. Connection to the App

**Figure 5:**
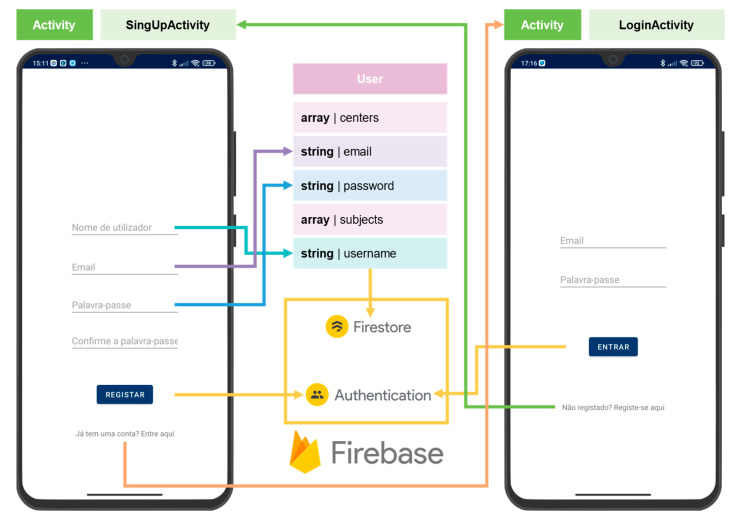
Data structures of the Wearables Balance app.

The User class is created in the signup window of the app, as shown in Figure 5.

The user must introduce the (i) username; (ii) email; and (iii) password (two-step verification). The email and password are used to create a new user in the Authentication service of Firebase. The Authentication service attributes a UID to the user, which names the corresponding document in the “users” collection of Cloud Firestore, storing the user’s/User object data.

The user must introduce the username and password in the login window to authenticate. The app connects to the Authentication service to sign-in/login. Despite the Wearables Balance app only allowing the email and password sign-in method, the Firebase’s Authentication service offers multiple sign-in methods, including via smartphone or other providers such as Google and Microsoft.

The signup and login procedures have a dedicated Activity for each other, respectively, SignUpActivity and LoginActivity. If the user already has an account, it is possible to navigate to the LoginActivity by selecting “Already has an account”. On the contrary, if the LoginActivity is active, the user can navigate to the SignUpActivity by selecting “Not registered?”.

#### 4.1.4. Navigating through the Wearables Balance App

**Figure 6:**
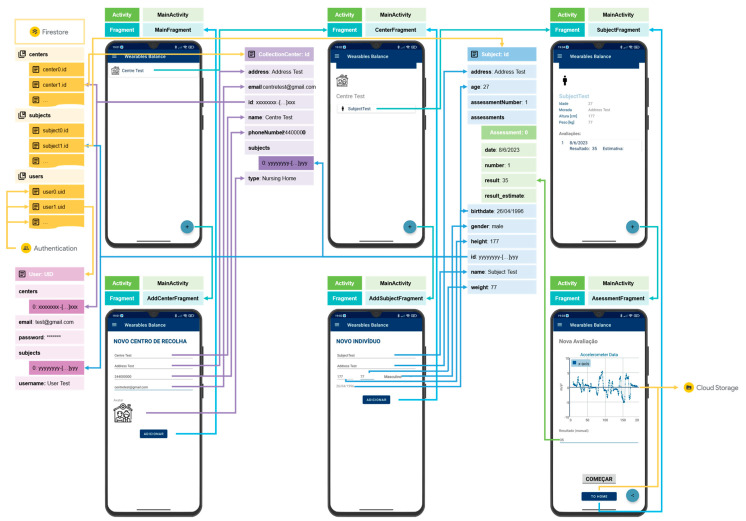
MainActivity structure and navigation: 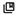 Cloud Firestore collection; 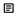 Cloud Firestore document.

Apart from signup and login frameworks, the app is fully contained in a single activity—MainActivity. This activity accounts for multiple fragments, as represented in Figure 6.

1. MainFragment—contains the app’s first screen and presents the list of collection centers in the user’s repository; the user can select a collection center to navigate to the CenterFragment.
2. CentersFragment—allows users to search for a collection center; the user can select a collection center to navigate to the CenterFragment.
3. CenterFragment—presents information about a collection center and the assigned subjects; the user can select a subject to navigate to the SubjectFragment.
4. SubjectFragment—displays the information about the selected subject, including the list and registry of the assessments performed on the subject.
5. AssessmentFragment—allows the user to perform an assessment, displaying the data of a selected axis of a selected sensor in a graph; the user can register the values obtained through the manual protocol and share the collected data through the shared platform to, for example, a cloud service (e.g., One Drive); the collected data is automatically uploaded to the cloud storage of Firebase when exiting the fragment.
6. AddCenterFragment—allows the user to add a new collection center, introducing the name, address, contact information, and type (home, nursing home, hospital, school, or other); a universally unique identifier (UUID) identifying the collection center is automatically generated; the fragment is accessed by pressing the add (“+”) button when the MainFragment or the CentersFragment is active.
7. AddSubjectFragment—allows the user to add a new subject, introducing the name and demographic data (birthdate, gender—male, female, or other –, weight, and height); a UUID identifying the subject is automatically generated; the fragment is accessed by pressing the add (“+”) button when the CenterFragment is active.

After login, the LoginActivity transits to the MainActivity, and the MainFragment will be active. The user data is obtained through a query on the Cloud Firestore “users” collection based on the UID generated when the respective account was created through the Authentication service. The “users” collection contains documents that store the data of each user. These documents are named after the UID attributed by the Authentication service. The centers’ data are stored in a “centers” collection in the Cloud Firestore, and, similarly to the “users” collection, the collection centers’ documents are named after the respective identifier.

Similarly, subjects’ data is stored in the “subjects” collection, and the documents are named after the respective subject identifier. A Manager class handles the information traffic related to the data structures. This class is responsible for querying to get data and update by uploading data to the Cloud Firestore collections.

The MainFragment view presents the list of collection centers in the user’s repository/centers array. This view contains an “Add” floating button (“+”) that allows the user to navigate to the AddCenterFragment. This fragment queries the user to enter the collection center information required to create a collection center. Pressing the “Add” button stores the data in a new CollectionCenter object, uploaded to the Cloud Firestore “centers” collection. The view returns to the MainFragment and, by selecting a collection center, the user can access the respective data, activating the CenterFragment. In this fragment, the App presents the list of subjects associated with the selected collection center. The process of adding a new subject is similar to the process of adding a new collection center. In the CenterFragment, when the user presses the “+” floating button, the AddSubjectFragment is activated. When the user presses the “Add” button after filling in the query, a new Subject object is created and uploaded to the “subjects” collection of the Cloud Firestore. Returning to the CenterFragment, the user can select a subject and navigate to the SubjectFragment, which presents the subject information and the list of assessments performed by the subject. In this fragment, pressing the “+” button navigates to the AssessmentFragment, which allows the user to perform a new assessment of the selected subject.

In the AssessmentFragment, the user can initiate a data collection process by pressing the “Start” button. When the process starts, the view presents a scatter graph that plots a sensor’s data in a selected axis. During the data collection process, the “Start” button changes the tag to “Stop”, allowing the user to end the process. The user can register the FRT’s manually obtained measurement and share the acquired data by the share button through the shared service. Additionally, when pressing the “To home” button to return to the SubjectFragment, the collected data is uploaded to the Cloud Storage service of the Firebase.

#### 4.1.5. Data Collection

Apart from the collected data, it is stored in the Cloud Storage in a folder whose name corresponds to the subject’s identifier. The data collected in the assessment are stored in ten (10) different text files (.csv extension):

1. id_assessmentNumber.csv: stores the data related to the assessment, namely the date of the assessment and the register of the result of the FRT obtained through the manual method;
2. id_assessmentNumber_acc.csv: accelerometer data in the tree axis (m∙s^−2^);
3. id_assessmentNumber_acc_r.csv: raw accelerometer data in the tree axis (m∙s^−2^);
4. id_assessmentNumber_gra.csv: a software-based estimate of the gravity acceleration in the tree axis (m∙s^−2^);
5. id_assessmentNumber_gyr.csv: gyroscope data in the tree axis (rad∙s^−1^);
6. id_assessmentNumber_gyr_r.csv: raw gyroscope data in the tree axis (rad∙s^−1^);
7. id_assessmentNumber_lin.csv: a software-based estimate of the linear acceleration (excluding gravity) in the tree axis (m∙s^−2^);
8. id_assessmentNumber_mag.csv: magnetometer data in the tree axis (μT);
9. id_assessmentNumber_mag_r.csv: raw magnetometer data in the tree axis (μT);
10. id_assessmentNumber_rot.csv: a software-based quaternion representing the device’s orientation (rotation vector sensor).

The first row of the files 2 to 10 presents the column labels of the document. The two first columns of the documents present the timestamp counting from the start of the assessment in milliseconds obtained through the System.currentTimeMillis function (system_ts_ms) and in nanoseconds (ns) obtained through the event.timestamp function (sensor_ts_ns). The following columns correspond to the acquired data in the identified axis (X, Y, and Z). The rotation vector data present an additional column corresponding to the magnitude of the quaternion (column labeled as “rotL”).

### 4.2. Orientation Estimation

MEMS sensor data was used to estimate the orientation of the device. Different estimation algorithms were tested and benchmarked against the software sensor provided by the Android sensor framework. The orientation algorithms tested were the complementary filter, EKF, Mahony algorithm, Madgwick algorithm, and our implementation of the Madgwick algorithm. The four first-named algorithms were available in the AHRS Python toolbox (v0.3) (https://github.com/Mayitzin/ahrs) (accessed on 18 December 2023).

The estimation of the orientation of the sensors array was computed using different algorithms available in the AHRS library of Python, as well as the Madgwick algorithm implemented in the context of the present paper. The algorithms were used to obtain a quaternion representation of the mobile device concerning the reference frame (Earth frame). Afterward, the Euler angles corresponding to the reference orientation quaternions and those computed through the tested algorithms were obtained. The obtained Euler angles were benchmarked against the estimate available by the Android library, which is considered the reference orientation. All tested algorithms can be computed in real time.

**Figure 7:**
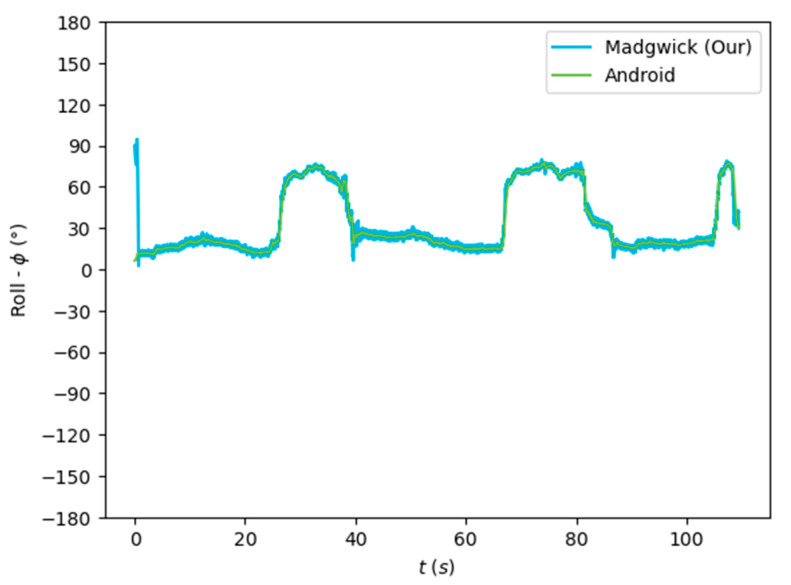
Euler angles (roll) obtained from the rotation vector from the software-based sensor and our implementation of the Madgwick algorithm.

**Figure 8:**
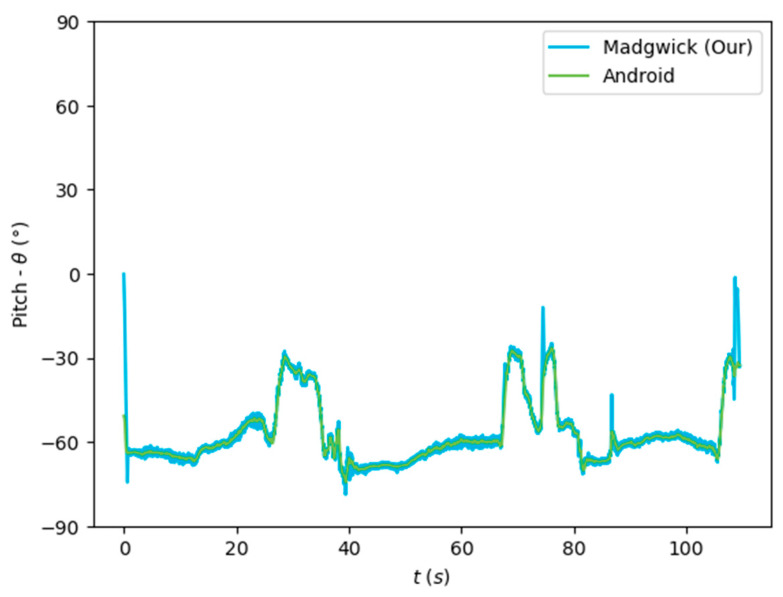
Euler angles (pitch) obtained from the rotation vector from the software-based sensor and our implementation of the Madgwick algorithm.

**Figure 9:**
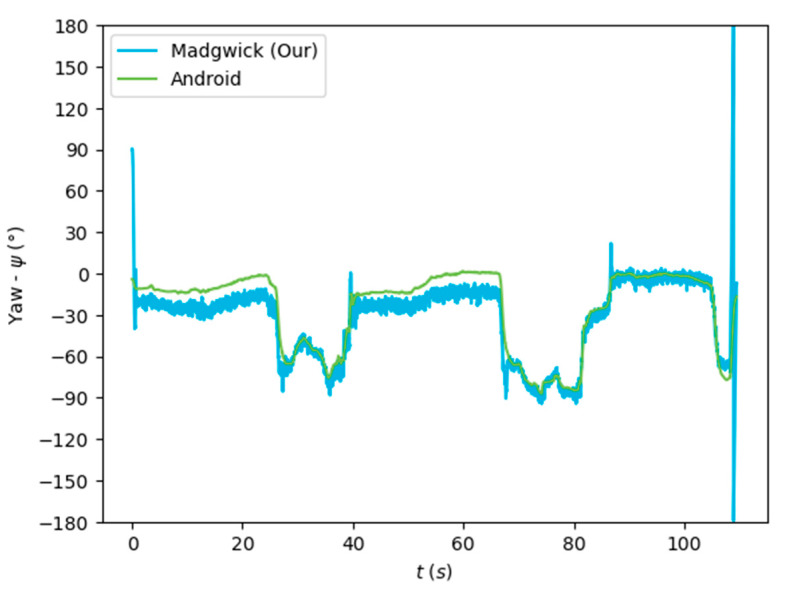
Euler angles (yaw) obtained from the rotation vector from the software-based sensor and our implementation of the Madgwick algorithm.

The algorithms were run on the complete dataset and were compared through an RMSE-based metric. Figure 7, Figure 8 and Figure 9 depict the Euler angles resulting from one of the trials in the acquired dataset. It was observed that the roll and pitch angles obtained with the tested algorithms corresponded to the Euler angles resulting from the software-based rotation vector made available by the Android Sensor package. However, the yaw angles obtained presented an offset of 90°, and thus, the Android-based angles were considered as described in Equation (19).

(19) $$\left[\begin{array}{c} \phi \\ \theta \\ \psi \end{array}\right]=\left[\begin{array}{c} \phi \\ \theta \\ \psi -90{}^{\circ} \end{array}\right]$$

The parameters described result in the Euler angles’ intervals described in Equation (20).

(20) $$\begin{array}{c} \phi \in \left[-180{}^{\circ},+180{}^{\circ}\right]\\ \theta \in \left[-90{}^{\circ},+90{}^{\circ}\right]\\ \psi \in \left[-180{}^{\circ},+180{}^{\circ}\right] \end{array}$$

**Figure 10:**
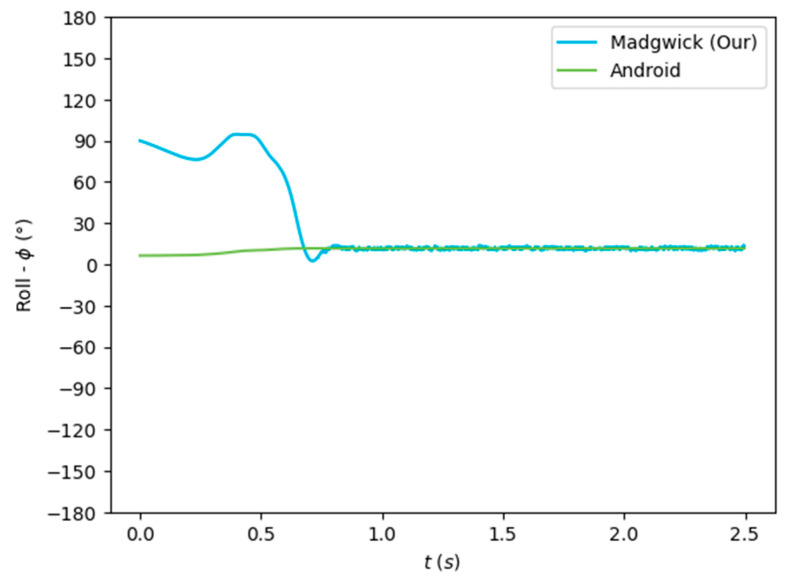
Convergence rate of the algorithm for the roll (*ϕ*) angle.

**Figure 11:**
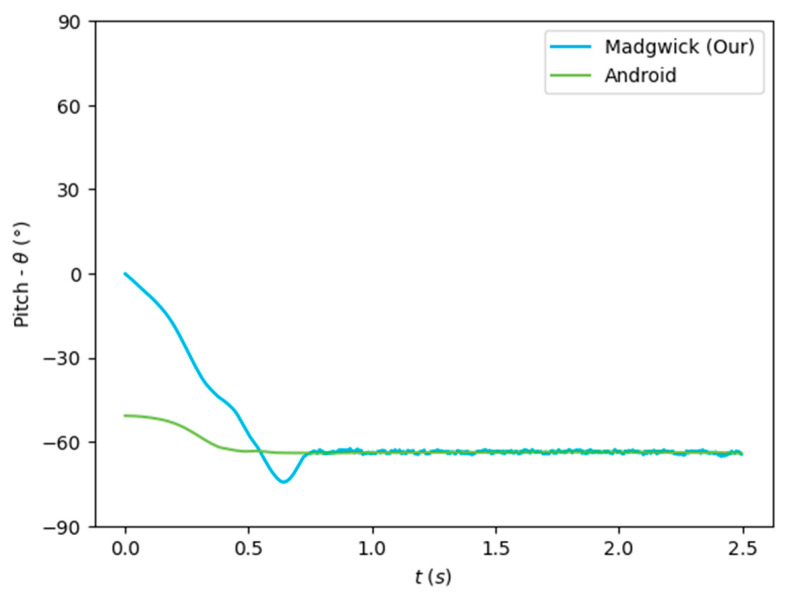
Convergence rate of the algorithm for the pitch (*θ*) angle.

Figure 7, Figure 8 and Figure 9 show that the algorithms performed reasonably well compared with the reference orientation (Android rotation vector). The transformation of the pitch angle, corresponding to the attitude, was, however, constant and corresponding to Equation (20). Moreover, taking a closer look at the beginning of the acquisition, it is possible to see that the algorithm’s convergence in the Roll and Pitch angles only takes a few data samples (less than 1 s), as shown in Figure 10 and Figure 11.

The orientation estimation results of the algorithms tested are presented in Table 3, compared to the Android SO data. The complementary filter obtained reasonably good results despite its low complexity. On the other hand, the EKF obtained a lower performance despite its greater complexity. The Mahony and Madgwick algorithms performed reasonably well, with the Mahony algorithm obtaining a better performance of the roll and pitch angles and penalizing the yaw angle.

Our implementation of the Madgwick algorithm presented the best results in the roll and pitch angles, as verified by the lower RMSE. The tests performed without magnetometer information resulted in considerable errors in the yaw angle, which was highly improved when using the data from this sensor. This algorithm, detailed in Section 3.2.2, was implemented in the developed mobile application (Java/Android) to obtain the real-time orientation estimate.

### 4.3. Position Estimation

Pose estimation considers the sensor’s position and the respective orientation. After obtaining the device’s orientation, the objective was to estimate the respective position to allow the computation of the FRT metric. The position was determined through the double integration of the linear acceleration. However, the accelerometer is sensible to linear acceleration and gravity. The sensor data was rotated from the sensor frame to the Earth frame (ENU) to remove the gravity component. This rotation allows the prompt remotion of the gravity component from the accelerometer data.

After obtaining the linear acceleration, the double integration should return the position of the sensor. However, due to noisy data, the integration led to considerable drift. An example using FRT data is presented below, showing the previous steps. The example is from an individual who performs 2 extension-and-return consecutive movements (2 consecutive FRT tentatives). The authors measured and registered the FRT reach for each movement with the ruler attached to the wall.

**Figure 12:**
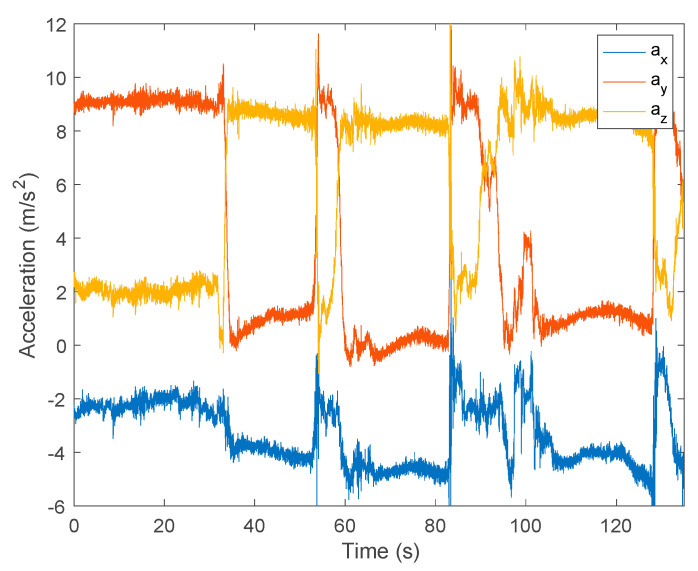
Raw acceleration data in the three component axes from an FRT test.

Figure 12 illustrates the raw acceleration data in the three axes (ax, ay, az) acquired by the accelerometer for an FRT data example.

**Figure 13:**
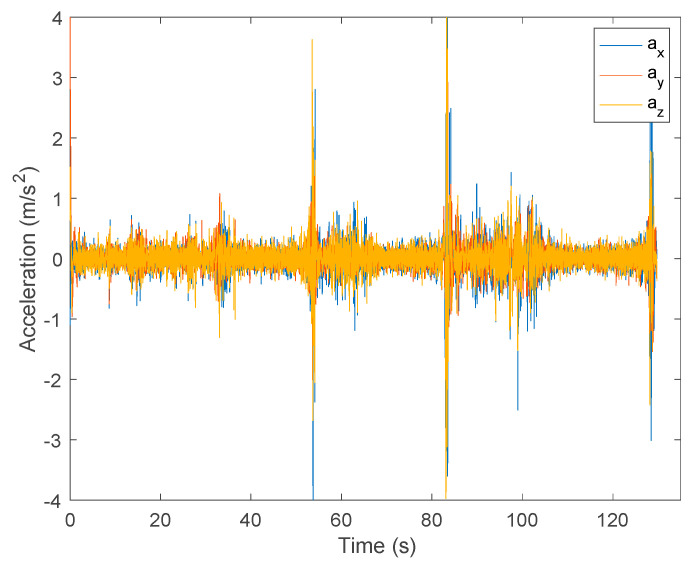
Filtered acceleration data in the three component axes from an FRT test.

The analysis of the acceleration data captured by the accelerometer in three directions (ax, ay, az) revealed a non-uniform time sampling around 5 ms. To address this irregularity, it was decided to resample the initial collected data using spline interpolation, resulting in a fixed sampling frequency of 100 Hz. The result of this resampling step presents a similar representation of data to that in Figure 13.

The presence of gravitational acceleration in the raw acceleration data required the application of a filter to isolate the subject’s motion information. A high-pass finite impulse response (FIR) filter of order 1000 with a cutoff frequency of 1 Hz was applied. This choice was justified by efficiently eliminating the gravitational component while preserving the acceleration information associated with the subject’s movement. Figure 13 presents the output of this filter.

**Figure 14:**
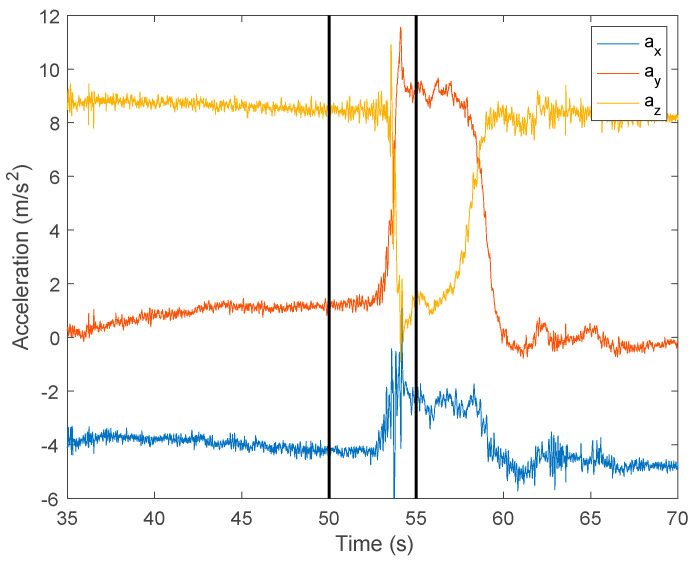
Highlight of the second intervals of the movement.

Afterwards, in the preprocessing phase, it was necessary to identify the specific time intervals with the subject’s movement. These intervals were obtained considering a constant interval of 5 s centered around each movement. Figure 14 highlights a section of Figure 12, demonstrating the methodology for determining the second interval for analysis (the end of the extension and return movement for the 1st FRT measurement of this test), resulting in a time interval from 50 to 55 s for this particular example.

### 4.4. Functional Reach Test Measurement

FRT metric estimation depended on appropriately estimating the mobile device’s position (and orientation). However, the determination of the FRT metric would consist of: (i) considering the XOY plane; (ii) obtaining the principal direction of the movement; and (iii) computing the maximum traveled distance in the obtained direction. Considering the XOY plane is straightforward since, if correctly implemented, the proposed methodology provides the mobile device pose in the Earth frame. Thus, the XOY plane would correspond to the position’s two first coordinates of the ℝ^3^ representation. The estimation of the principal direction of the movement can be obtained by allowing the computation of the FRT metric. Table 4 presents the results for five individuals for two trials each.

By integrating the acceleration information in Table 4, it is possible to estimate velocity and, subsequently, the displacement of the subject by integrating the velocity information. The estimated displacements, also depicted in Table 4, range from 11.45 cm to 27.09 cm.

Table 5 depicts data on the functional reach test for five individuals, comparing estimated vs. measured displacement in centimeters and calculating the error between these measurements. The average estimated displacements range from 12.62 cm to 22.06 cm, whereas the average measured displacements vary from 16.90 cm to 23.10 cm. The error in displacement measurements indicates discrepancies between estimated and actual performance, with values ranging from 1.22 cm to 6.28 cm, showcasing individual variability in estimating and achieving reach distances.

Additionally, the estimated measurements are in an acceptable range compared to those made in situ by the authors to grant a valid ground truth measurement.

## 5. Conclusions

Our study focuses on assessing and improving balance in older adults, with the ultimate goal of reducing the occurrence of falls. To achieve this, we are developing an innovative automated tool designed explicitly for evaluating balance in older adults. This tool utilizes MEMS technology found in commonly used smart wearables and smartphones, enabling the computation of the FRT metric.

To estimate the FRT, our proposed method employs the Madgwick orientation algorithm, which utilizes quaternion algebra to determine the sensor’s orientation in relation to the Earth’s frame of reference. By applying this algorithm, it is possible to eliminate the influence of gravity from the accelerometer data. These data are then integrated twice to determine the sensor’s position. By measuring the distance traveled by the sensor in the XOY plane, it is possible to determine the FRT metric, which provides valuable insights into an individual’s balance.

Regarding similar and comparable works, The G-STRIDE system [76], an inertial-sensor-based gait analysis tool, is examined to determine the likelihood of falls in this population. It focuses on particular gait factors, including total distance, velocity, and cadence, to identify those more likely to fall. It uses a multifactorial approach that includes clinical examination and gait analysis. Yoon et al. [77] explored using IMU sensors for gait analysis across different age groups. The study found significant differences in gait parameters and joint angles between adults and older adults, underlining the impact of aging on gait characteristics and the utility of IMU sensors in capturing these changes. Hina Shafi et al. [78] utilized smartphone applications to assess gait and balance in adults with mild balance impairment. The study demonstrated these apps’ reliability, validity, and sensitivity in measuring various gait and balance parameters, highlighting their potential as accessible tools for health assessments in low-resource settings. Although the applications are somewhat different, they focus on using mobile and sensor technologies in health assessments, similar to our work, highlighting the potential of inertial sensors in clinical settings to improve fall risk assessment.

This paper’s primary outcome was deploying a new tool to collect data from mobile devices during an FRT assessment to prepare a database with the collected data. Afterward, an orientation estimation algorithm based on inertial data was implemented. The reasonable performance of the orientation estimation algorithms enabled a proper removal of the gravity component from the accelerometer data.

In future work, due to the sensor noise, the position estimation must be improved with further methods to reduce the sensors’ drift integration.

## Figures and Tables

**Table 1:** Functional reach test thresholds (cm) for considerable fall risk in different age groups [13].

Age (Years)	Men	Woman
20–40	42.49 cm	37.19 cm
41–69	38.25 cm	35.08 cm
70–87	33.43 cm	26.59 cm

**Table 2:** Advantages and disadvantages of different representations of rotations.

Methods	Advantages	Disadvantages
Rotation Matrices	Matrix presentation for a single rotation; Matrix operations are well-known; Make calculations easier; Homogeneous matrices represent all the basic transformations.	Nine degrees of freedom; Six orthogonality constraints; Risk of rounding errors in the successive concatenation of matrices; The rotation matrix is intricate to construct when the base of the space in which the rotation is performed is not known; Interpolation is problematic.
Euler Angles	Three degrees of freedom; Intuitive method; Natural and efficient representation; Simple form for rotations around coordinate axes	There is not always a straightforward decomposition of the rotation into three rotations around the coordinate axes; There are 12 different ways to compose elemental rotations; The representation of concatenated rotations is quite complex; Possible loss of one degree of freedom—Gimbal Lock problematic interpolation
Axis-Angle	Four degrees of freedom; good visualization	Possible loss of unitary norm; Numerical errors can affect the angle value Computational difficulty in composing rotations; Ambiguity in choosing axis orientation; Multiplicity of identity representation; Problematic interpolation
Quaternions	Four degrees of freedom; Simplicity and economy; Ease of combining rotations; The choice of the coordinate system does not influence.	Indetermination in the orientation of the axes: q and −q represent the same rotation; Represent only rotations. Unintuitive and challenging to visualize.

**Table 3:** Benchmark of Euler angles obtained from different AHRS algorithms, using the root mean squared error (RMSE).

Algorithm	Roll (*ϕ*)	Pitch (*θ*)	Yaw (*ψ*)
Complementary Filter	9.4	1.6	34.7
Extended Kalman Filter	91.4	24.1	96.4
Mahony	10.8	0.9	93.5
Madgwick	24.2	3.7	55.3
Madgwick (ours, without magnetometer)	8.6	0.9	119.1
Madgwick (ours, complete)	10.3	1.1	38.1

**Table 4:** Two complete trial FRT movement data for five individuals: calculated and measured horizontal displacement.

Individual	First FRT Trial		Second FRT Trial	
	Estimated Displacement (cm)	Measured Displacement (cm)	Estimated Displacement (cm)	Measured Displacement (cm)
1	17.02	14.00	27.09	25.50
2	19.45	23.40	14.20	22.80
3	14.06	18.10	19.00	17.40
4	11.45	14.50	13.79	19.30
5	11.65	16.30	17.75	21.60

**Table 5:** Comparison of average estimated and measured FRT displacements in five individuals.

Individual	Average Estimated Displacement (cm)	Average Measured Displacement (cm)	Average Displacement Error (cm)
1	22.06	19.75	2.31
2	16.83	23.10	6.28
3	16.53	17.75	1.22
4	12.62	16.90	4.28
5	14.70	18.95	4.25

## Data Availability

Data will be available by request to the corresponding author.
